# Air of the Dog: Impending Respiratory Failure After a Dog Bite

**DOI:** 10.7759/cureus.4304

**Published:** 2019-03-23

**Authors:** Gregory Tanquary, Andrew Little, Juan A Morales-Torres

**Affiliations:** 1 Emergency Medicine, Ohiohealth Doctors Hospital, Columbus, USA; 2 Emergency Medicine, Ohiohealth Doctors Hospital, Columbus , USA

**Keywords:** dog bite, penetrating neck trauma, respiratory failure

## Abstract

Animal and human bites represent a significant health issue worldwide. However, penetrating neck trauma secondary to animal bites is a rare presentation. We present the case of a 32-year-old female with neck pain and difficulty breathing after suffering a dog bite to the neck. Bedside ultrasound and subsequent computed tomography (CT) showed findings consistent with subcutaneous emphysema. Due to rapidly progressing neck swelling and stridor, she required advanced airway management followed by emergent exploratory surgery. We present a case report on impending respiratory failure after a dog bite and review the management of penetrating neck trauma.

## Introduction

Animal and human bites represent a significant health issue worldwide. Sixty-five percent of United States households, or about 79.7 million families, own a pet, according to the 2015-2016 National Pet Owners Survey conducted by the American Pet Products Association [1]. This is up from 56% of U.S. households in 1988, the first year the survey was conducted [1]. Furthermore, approximately 4.5 million dog bites occur each year in the U.S. [2]. This results in 800,000 Americans bitten by dogs each year, 386,000 of which require treatment in emergency departments [3].

The majority of dog bite investigations involve pediatric populations due to an increased incidence. Based on reports by the National Center for Injury and Prevention, dog bite injuries in pediatric patients occurred most commonly to the arm/hand (45.3%), leg/foot (25.8%), and head/neck (22.8%) [2]. This is in contrast to adult injuries in which only 10% of bites involve the head and neck [4]. While rare, this does create the potential for lethal complications, including vascular and respiratory compromise in adult dog bite victims. In a retrospective review of patients treated in a jail ward emergency department after suffering K9-related injuries, vascular injuries were sustained in 49 patients (7.0%), 35 (5.0%) sustained infections, 28 (4.0%) sustained open fractures and cortical violations, 13 (1.9%) sustained nerve injuries, eight (1.1%) sustained tendon injuries, and six (.9%) sustained open joints [5]. While vascular and infectious complications are relatively well-studied, airway management from these injuries has been less extensively investigated.

## Case presentation

Most research regarding dog bites has been devoted to the surgical management of pediatric injuries. The need for this research is supported by the fact that dog bites are more prevalent in the pediatric population (Table 1). A database maintained by the Agency for Health Care Policy and Research found that the incidence of dog bite cases is inversely related to patient age [6]. However, research surrounding pediatric dog bites does not focus on airway management by the emergency physician. Our case illustrates the potential severity of dog bites. More specifically, this encounter highlights the indications and low threshold an emergency physician must have in airway management secondary to penetrating neck trauma.

**Table 1:** Dog bite discharge rates per 100,000 according to the Health Care Utilization Project (HCUP)

Age (years)	Incidence per 100,000 people
0 -4	5.0
5 - 9	4.9
10 - 14	2.6
15 – 19	1.2
20 – 39	1.6
> 40	2.0

A 32-year-old, previously healthy, female presented to the emergency department with the chief complaint of neck pain after suffering a dog bite. Upon arrival, she began to complain of neck swelling and difficulty speaking. Notably, she reported trouble breathing and it became obvious she was struggling to manage her secretions. She stated her symptoms were progressively worsening. She had no other injuries to report. On a review of systems, the patient did endorse odynophagia, dysphagia, trismus, near-syncope, and dyspnea.

On initial examination, our patient was anxious, tripoding, and in obvious distress. Her voice was softened and hoarse. She had two separate lacerations to her neck, both in "Zone 2." The first was a two-centimeter linear laceration located immediately lateral to the left common carotid. It appeared to involve only the dermis and was not actively bleeding. The second was a one-centimeter linear laceration located over the right sternocleidomastoid (SCM) muscle, also superficial and not actively bleeding. Neither site was probed, to avoid an iatrogenic neurovascular injury. Obvious swelling to the anterior and left lateral neck was appreciated. There was no tracheal deviation. The posterior oropharynx could not be visualized secondary to pain and limited jaw mobility. Respirations were labored, with lung sounds clear to auscultation. No stridor was heard. A notable hard sign of a vascular injury that was present included rapidly expanding hematoma. Furthermore, two soft signs of vascular injury included hoarse voice and subcutaneous emphysema. The remainder of the examination was unremarkable.

**Figure 1:**
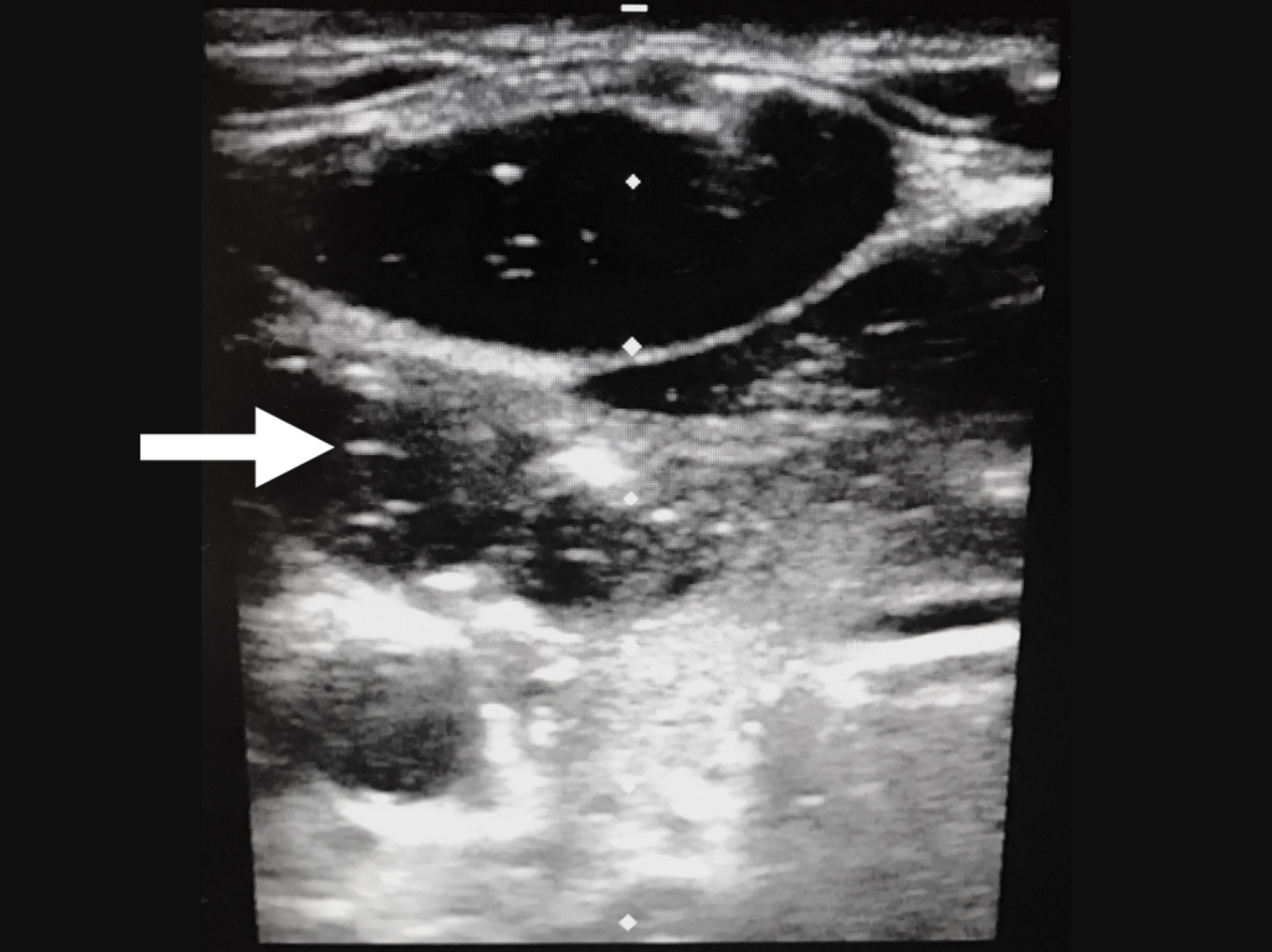
Ultrasound (US) image of the area around the patient’s largest bite wound A large number of air streaks/artifacts (scattered white horizontal lines, as shown by the white arrow) and an expanding subcutaneous hematoma are visible.

Airway management was paramount given the patient’s concerning respiratory status, including accessory muscle use, dysphonia, limited jaw mobility, and obvious signs of trauma to the anterior neck. While preparing for a definitive airway, the patient underwent bedside ultrasound of her neck. Images obtained using point of care ultrasound (Figure 1) showed multiple areas of free air, along with a left lateral neck wall hematoma.

Etomidate and rocuronium were used for sedation and paralysis, respectively, to perform rapid sequence intubation. Glidescope was utilized for intubation given the suspected difficult airway. During endotracheal intubation, vocal cords were visualized with mild surrounding edema. No obvious signs of tracheal violation were seen, including hemorrhage.

**Figure 2:**
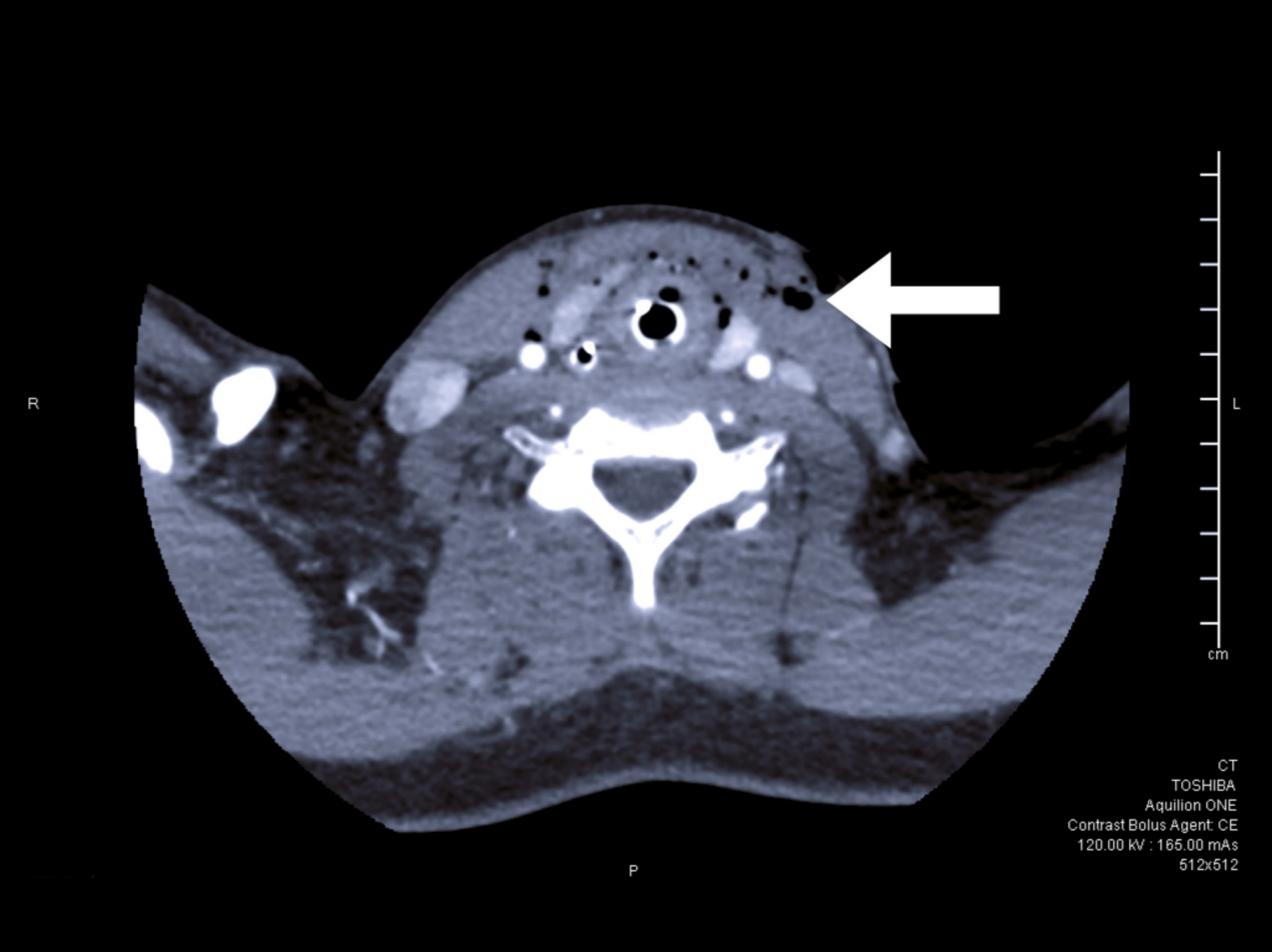
Soft tissue computed tomography of the neck after intubation, showing deep space air from the dog bite (as seen by the arrow)

The patient did receive a tetanus vaccine update as cephalosporin antibiotic administration. The wounds to the neck were covered with bandages and secured. After the patient was stabilized, she was transported to the nearest Level 1 trauma center where advanced imaging was performed. Computed tomography (CT) cervical spine without contrast and CT angiogram of the neck were ordered. CT neck demonstrated anterior neck swelling with numerous foci-of-emphysema in the anterior neck soft tissues (Figure 2). No active extravasation of contrast was seen.

Trauma surgery documented that more subcutaneous air was seen on imaging than to be expected, given the external appearance of her wounds. The patient was taken to the operating room (OR) for exploratory surgery with intraoperative EGD. Intraoperative findings included a laceration to the left SCM muscle, which violated the platysma and tunneled deep into the soft tissue. No signs of tracheal, esophageal, or vascular injury were seen. The right neck wound was also explored and revealed deep penetration as well, terminating at the tracheoesophageal groove. Intraoperative EGD did not reveal any penetrating wounds to the GI tract. Postoperatively, a Penrose drain was kept in place through the patient’s left neck wound. She was started on ciprofloxacin and clindamycin secondary to a penicillin allergy. The patient was extubated the following day without complication.

The remainder of her hospital course was uneventful. The patient was discharged with ciprofloxacin and clindamycin.

## Discussion

Our case illustrates how a benign-appearing wound could progress to impending respiratory failure without intervention. In a retrospective case review over the course of five years at the University of New Mexico Health Sciences Center, 16 severely injured children (36%) were hospitalized to repair damage to their airway and blood vessels of the neck or to treat hemodynamic compromise from dog bites to the face, scalp, or head. Although uncommon, when dog bite injuries do occur to the face or neck, emergent airway complications can develop.

One of the first studies to document criteria for emergent intubations was by Eggen and Jorden. They described 69 intubations, including 29 emergency department intubations (seven nasal intubations, nine oral intubations, eight tracheostomies, four wound intubations, and one unspecified case). Criteria for intubation included acute respiratory distress, airway compromise from blood or secretions, extensive subcutaneous emphysema, tracheal shift, or severe alteration in mental status [7].

As illustrated by our case, a low threshold for intubation is important in acute respiratory distress, particularly in the setting of penetrating neck trauma with subcutaneous emphysema. The mechanism of injury, physical exam findings, and clinical picture of the patient should all be considered when determining the need for intubation. It is important to intubate patients with high-risk airways early to prevent a more difficult endotracheal intubation from swelling, secretions, or bleeding. Furthermore, this may prevent the need for surgical airway management, carries a risk of lower success rates and higher complications.

## Conclusions

Our case highlights how a dog bite that appears simple can violate deeper structures in the neck, leading to airway compromise. When evaluating and treating neck injuries, especially from dog bites, it is necessary to maintain a high index of suspicion for a major underlying injury. Early intubation is key in high-risk airways to increase chances of successful endotracheal intubation and decrease complication rates.
